# A rare case of alopecia universalis

**DOI:** 10.11604/pamj.2023.46.44.41181

**Published:** 2023-09-29

**Authors:** Amol Deshpande, Mayuri Deshpande

**Affiliations:** 1 Department of Rachana Sharir, Mahatma Gandhi Ayurved College and Research Centre, Datta Meghe Institute of Higher Education and Research (Deemed to be University) Salod (H), Wardha, Maharashtra, India,; 2 Department of Kayachikitsa, Mahatma Gandhi Ayurved College and Research Centre, Datta Meghe Institute of Higher Education and Research (Deemed to be University) Salod (H), Wardha, Maharashtra, India

**Keywords:** Alopecia universalis, alopecia areata, alopecia totalis

## Image in medicine

A 28-year-old patient came to the outpatient department of Mahatma Gandhi Ayurved College Hospital and Research Center, Salod, Wardha with complete loss of hairs from all over the body. First, he had Alopecia areata, but then he developed complete hair loss. He took various medications but of no use. Alopecia universalis is a rare variation of alopecia areata (AA), characterized by widespread hair loss that affects both the scalp and the body. Inhibitors of the tumor necrosis factor (TNF) have mainly failed to treat AA and have been found to either cause or exacerbate AA in some people. Unknown is the precise pathophysiology of AA. It is thought that stress, environmental circumstances, and genetics all play a part. Perifollicular inflammation and, in particular, a peribulbar lymphocytic infiltration surrounding anagen hair follicles can be seen in pathology specimens from patients with active illness. Inflammation, changes in hair cycling, and eventually hair loss are thought to result from CD4+ and CD8+ T lymphocytes infiltrating the hair and becoming reactive to hair bulb autoantigens.

**Figure 1:**
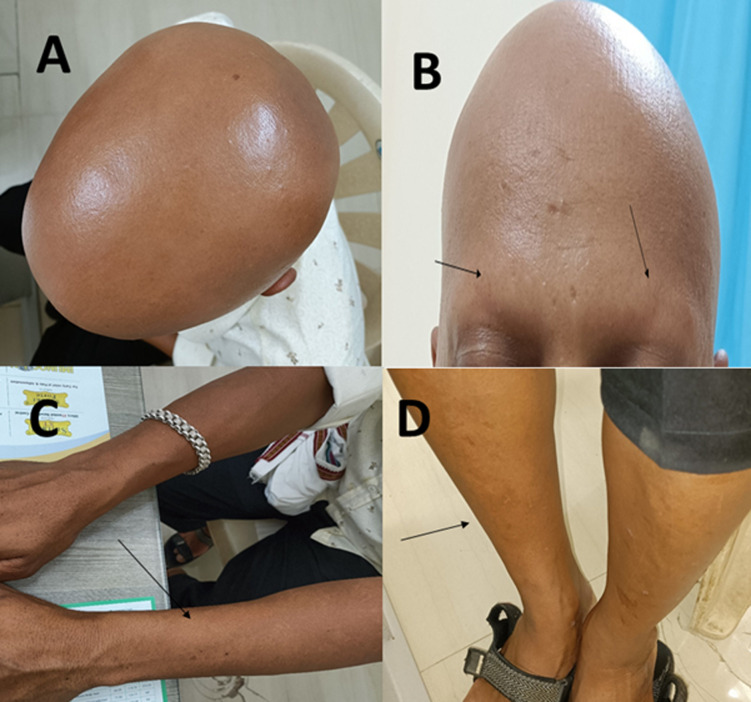
A) complete hair loss on the head; B) no eyebrows; C) hairless hands; D) hairless legs

